# A dual-database bibliometric analysis on dry needling for pain: Global trends and hotspots from web of science core collection and Scopus (2006–2025)

**DOI:** 10.3389/fneur.2026.1713196

**Published:** 2026-03-04

**Authors:** Mo Liao, Xinhui Cheng, Fei Liu, Xingjuan Xiong, Yongsheng Yu

**Affiliations:** Rehabilitation Center, Dazhou Integrated TCM & Western Medicine Hospital, Dazhou, Sichuan, China

**Keywords:** bibliometrics, clinical trial design, evidence-based medicine, international collaboration, myofascial pain

## Abstract

**Objective:**

Dry needling (DN) has been shown to exert beneficial effects in pain management. However, a comprehensive bibliometric analysis specifically examining the relationship between DN and pain has not yet been conducted. This study aims to systematically evaluate the global research landscape and emerging trends in the field of “dry needling and pain” from 2006 to 2025 using bibliometric methods, thereby providing a quantitative foundation and forward-looking guidance for future basic and clinical research.

**Methods:**

A bibliometric analysis was conducted using literature published between 2006 and 2025, retrieved from the Web of Science Core Collection (WoSCC) and Scopus databases. Data were analyzed with Bibliometrix (R package), VOSviewer, and CiteSpace software.

**Results:**

A total of 936 and 1,149 articles were retrieved from the WoSCC and Scopus databases, respectively. Over the past two decades, publication output in this field has steadily increased. The United States and Spain were the leading contributing countries, with Universidad Rey Juan Carlos in Spain serving as a central hub within the international collaboration network. Fernández-De-Las-Peñas C. was identified as the most prolific author. Journal analysis showed that the Journal of Bodywork and Movement Therapies published the most articles, whereas the Archives of Physical Medicine and Rehabilitation received the highest number of citations and acted as a key node in scholarly cooperation. Keyword co-occurrence and clustering analyses revealed four core research themes: evidence-based pain management, pathophysiological mechanisms of myofascial trigger points (MTrPs), clinical efficacy of DN for myofascial pain, and clinical trial design with outcome assessment. Current research hotspots focus on clinical effectiveness, mechanistic studies, refinement of trial methodologies, standardization of outcomes, and risk management in clinical practice.

**Conclusion:**

The application of DN in pain management has garnered increasing global attention and is poised to become a major focus within the field. This study provides a comprehensive overview of the current research status and emerging themes, offering valuable insights for future investigations.

## 1 Introduction

Pain is one of the most prevalent health problems worldwide and a major contributor to functional impairment, reduced quality of life, and increased healthcare utilization (1–3). With the continuously rising prevalence of chronic pain, the identification of safe, effective, and reproducible non-pharmacological interventions has become a key research priority in contemporary pain management (4, 5). Dry needling (DN), a minimally invasive technique targeting myofascial trigger points (MTrPs), modulates local muscle tension and nociceptive signaling through direct mechanical stimulation. It has demonstrated therapeutic potential in the management of various musculoskeletal pain conditions, including neck–shoulder pain and low back pain, and has accumulated an expanding body of clinical and mechanistic evidence (6–17). As the volume of research in this field has increased rapidly, the application of systematic quantitative approaches to delineate its developmental trajectory and to identify knowledge structures and collaboration networks has become increasingly important for understanding disciplinary trends and guiding future research.

Bibliometric analysis is a quantitative methodology widely used to elucidate disciplinary structures, research trends, and the evolution of scientific knowledge, and has been increasingly applied in pain medicine and rehabilitation research (18). Although several bibliometric studies have examined acupuncture, myofascial pain, or non-pharmacological analgesic strategies, most have either combined DN with other needling techniques, thereby obscuring its specific technical characteristics and research pathways, or relied on a single database, which limits a comprehensive assessment of the global research landscape.

Given the differences between the Web of Science Core Collection (WoSCC) and Scopus in journal coverage, disciplinary emphasis, and citation indexing strategies, the combined use of these two databases with parallel analyses may provide a more comprehensive and nuanced understanding of the global knowledge structure and developmental dynamics of DN research for pain. Therefore, a multi-database bibliometric approach is warranted to systematically examine research output, collaboration networks, knowledge bases, and research hotspots in this field, thereby providing an evidence-based reference for future study design and academic development.

As the potential mechanisms by which dry needling exerts analgesic effects involve neural responses and modulation of pain signaling, a bibliometric analysis of this field can also capture and reflect research trends and accumulated knowledge related to pain management at the neuromuscular level. Accordingly, the present study aims to conduct a systematic bibliometric and visualization analysis of research on DN interventions for pain. We hypothesize that the research evolution in this field follows a discernible progression from mechanistic exploration to clinical efficacy evaluation, and subsequently toward integrative treatment strategies and practice standardization, and that international collaboration networks exhibit a highly centralized structure dominated by a limited number of highly productive countries and institutions. By examining these hypotheses, this study seeks to offer empirical insights into the knowledge development patterns of this field and to inform future research directions.

## 2 Materials and methods

### 2.1 Data collection

**Figure 1:**
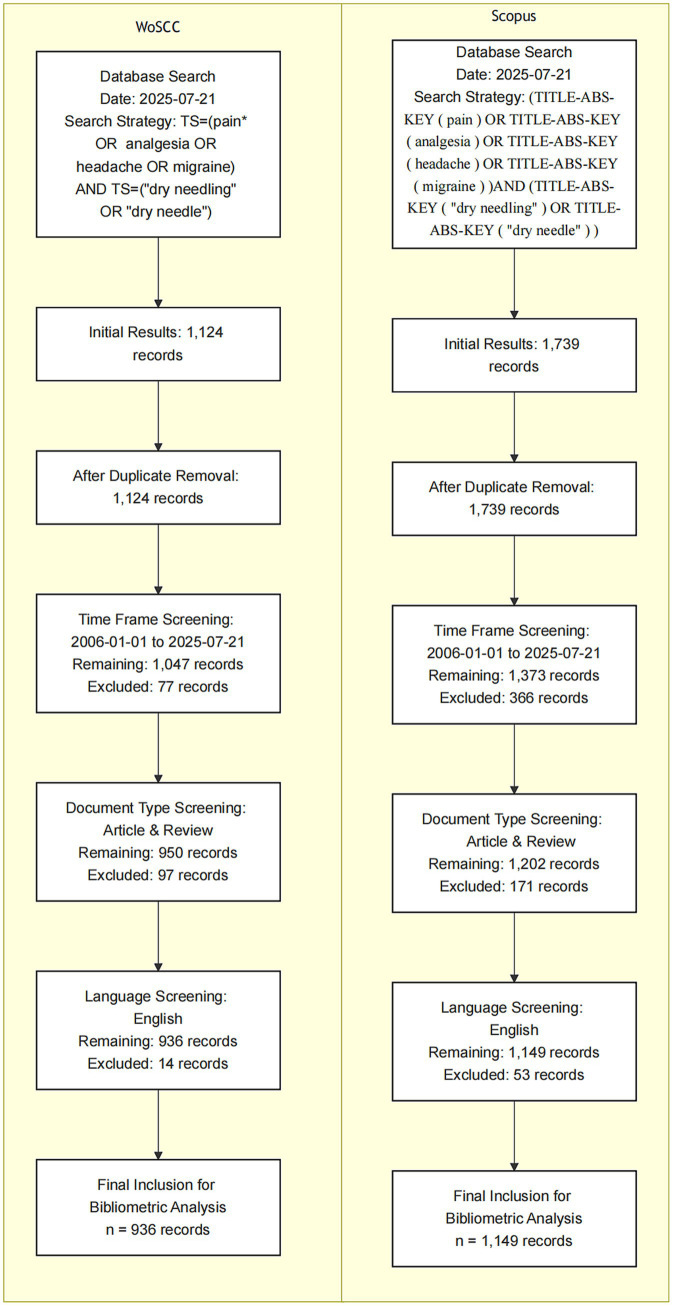
Literature search and screening process for studies on DN for pain in WoSCC and Scopus.

Data were retrieved from the WoSCC and Scopus, with the search conducted on July 21, 2025. In WoSCC, the following search strategy was applied: TS = (pain* OR analgesia OR headache OR migraine) AND TS = (“dry needling” OR “dry needle”). After excluding irrelevant records and restricting the publication period to January 1, 2006–July 21, 2025, document types to articles or reviews, and language criteria, a total of 936 records were identified, with duplicates removed. The retrieved records were saved in plain text format and exported as full records, including cited references. In Scopus, the search strategy was as follows: (TITLE-ABS-KEY (pain) OR TITLE-ABS-KEY (analgesia) OR TITLE-ABS-KEY (headache) OR TITLE-ABS-KEY (migraine)) AND (TITLE-ABS-KEY (“dry needling”) OR TITLE-ABS-KEY (“dry needle”)). After applying the same exclusion criteria and restrictions on publication period, document type (article or review), and language, 1,149 records were identified, with duplicates removed. These records were saved in CSV format and exported as full records, including cited references. The detailed selection process is illustrated in Figure 1.

### 2.2 Data analysis

In this study, we adopted a previously established methodological framework (19). A parallel analysis strategy was applied to the Web of Science Core Collection (WoSCC) and Scopus databases, whereby the two datasets were processed independently rather than merged. This design was based on two main considerations. First, the two databases differ in metadata structures, such as keyword field definitions and reference formats, and merging them may introduce mapping errors. Second, parallel analysis facilitates the assessment of the robustness of findings across different database environments, thereby making the potential influence of database selection on the results more transparent. All analytical procedures, including data cleaning, network construction, and burst detection, were conducted separately within two independent data streams, and the final results were validated through cross-comparison. Annual publication trends were analyzed using Origin 2018. In addition, R software (version 4.5.1) with the bibliometrix package (version 4.0)^1^ (20), VOSviewer (version 1.6.20) (21), and CiteSpace (version 6.1.4) (22) were employed for bibliometric analysis and visualization. The bibliometrix package was used to perform quantitative analysis and visual mapping of scientific knowledge. VOSviewer was applied to visualize co-authorship networks at the country and institutional levels, co-citation networks of source journals, and keyword co-occurrence patterns.

For analyses based on the WoSCC database, the minimum publication thresholds in co-authorship network analysis were set at ≥4 documents for countries, ≥4 for institutions, and ≥3 for authors. In co-citation analysis, source journals were required to have a minimum of 40 citations. For keyword co-occurrence analysis, the minimum occurrence frequency was set at ≥10. For analyses based on the Scopus database, the corresponding thresholds were ≥5 documents for countries, ≥4 for institutions, and ≥3 for authors in co-authorship network analysis; ≥40 citations for source journal co-citation analysis; and ≥30 occurrences for keyword co-occurrence analysis.

Threshold settings were determined based on established bibliometric practices and recommendations provided by VOSviewer. During network construction and visualization, node filtering was applied to reduce noise and highlight core structures. Setting minimum thresholds for co-citation counts and keyword occurrences is a common and necessary approach to identify representative literature and key terms and to enhance the interpretability of bibliometric analyses (21, 23). Keywords such as “dry needling,” “pain,” and their synonyms were excluded from the keyword analysis. CiteSpace was used for burst detection analysis with the following parameter settings: time slicing was set to one year per slice (time span: 2006–2025); the node type was set to “reference” for detecting reference bursts; and the selection criterion was based on the g-index with k = 25. Journal impact factors (IFs) for 2024 were retrieved from the Journal Citation Reports (JCR).

## 3 Results

### 3.1 Overview of research on DN and pain

**Figure 2:**
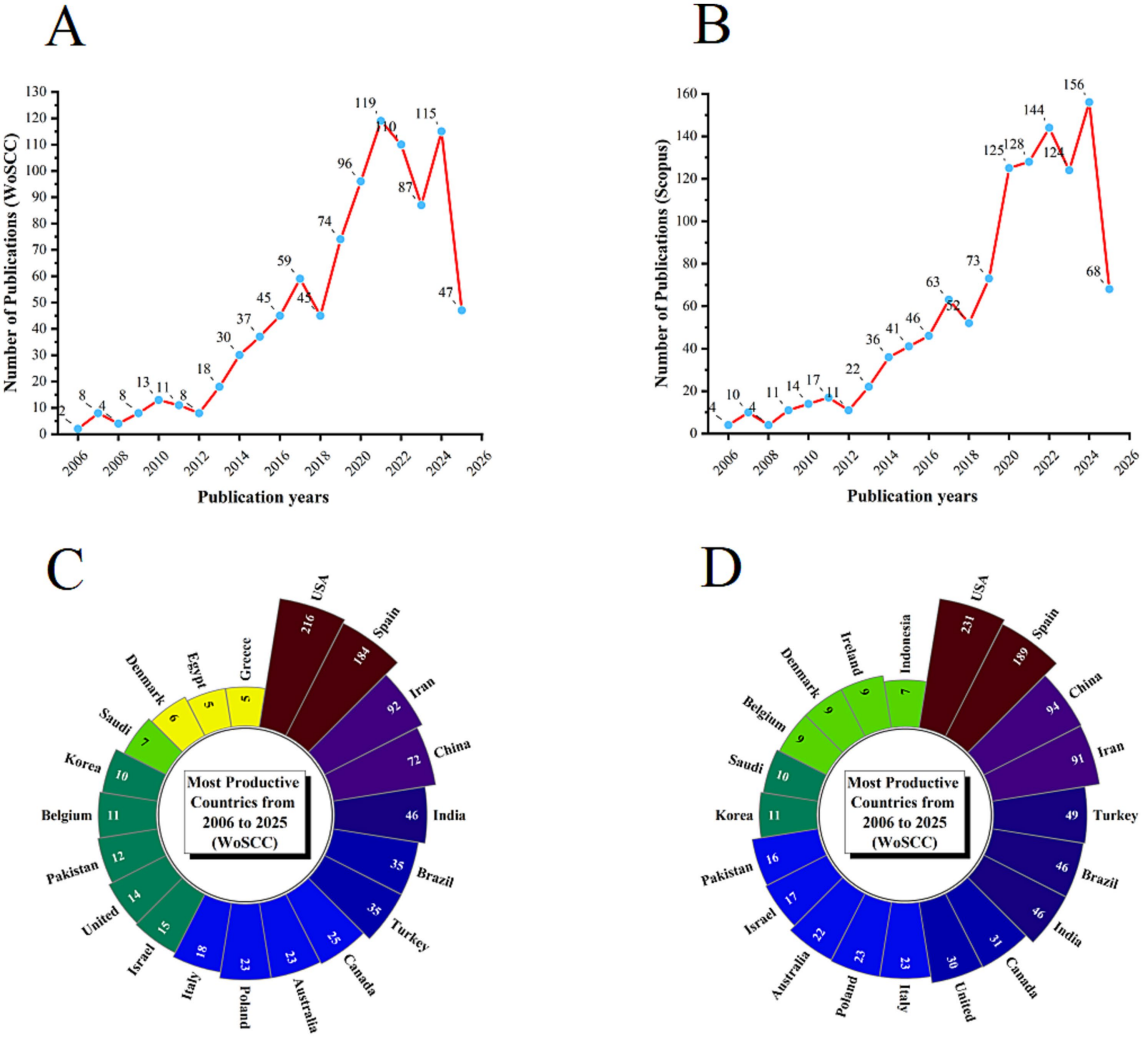
Annual publication trends on the relationship between DN and pain from 2006 to 2025: **(A)** Annual publication trends in WoSCC; **(B)** annual publication trends in Scopus; **(C)** distribution of corresponding authors’ countries in WoSCC; **(D)** distribution of corresponding authors’ countries in Scopus.

In the WoSCC database, a total of 936 unique records were identified after the removal of duplicates. From 2006 to 2021, the number of publications related to DN and pain exhibited a steady upward trend, followed by a slight decline in 2022 and 2023, and a resurgence in 2024, as illustrated in Figure 2A. In the Scopus database, 1,149 unique records were identified after duplicates were excluded. The publication count continued to rise from 2006 to 2022, experienced a slight decrease in 2023, and increased again in 2024, as shown in Figure 2B. The decline observed between 2022 and 2023 may be attributable to the impacts of the COVID-19 pandemic. The overall consistent growth across both databases underscores a rising global interest in exploring the relationship between DN and pain.

**Figure 3:**
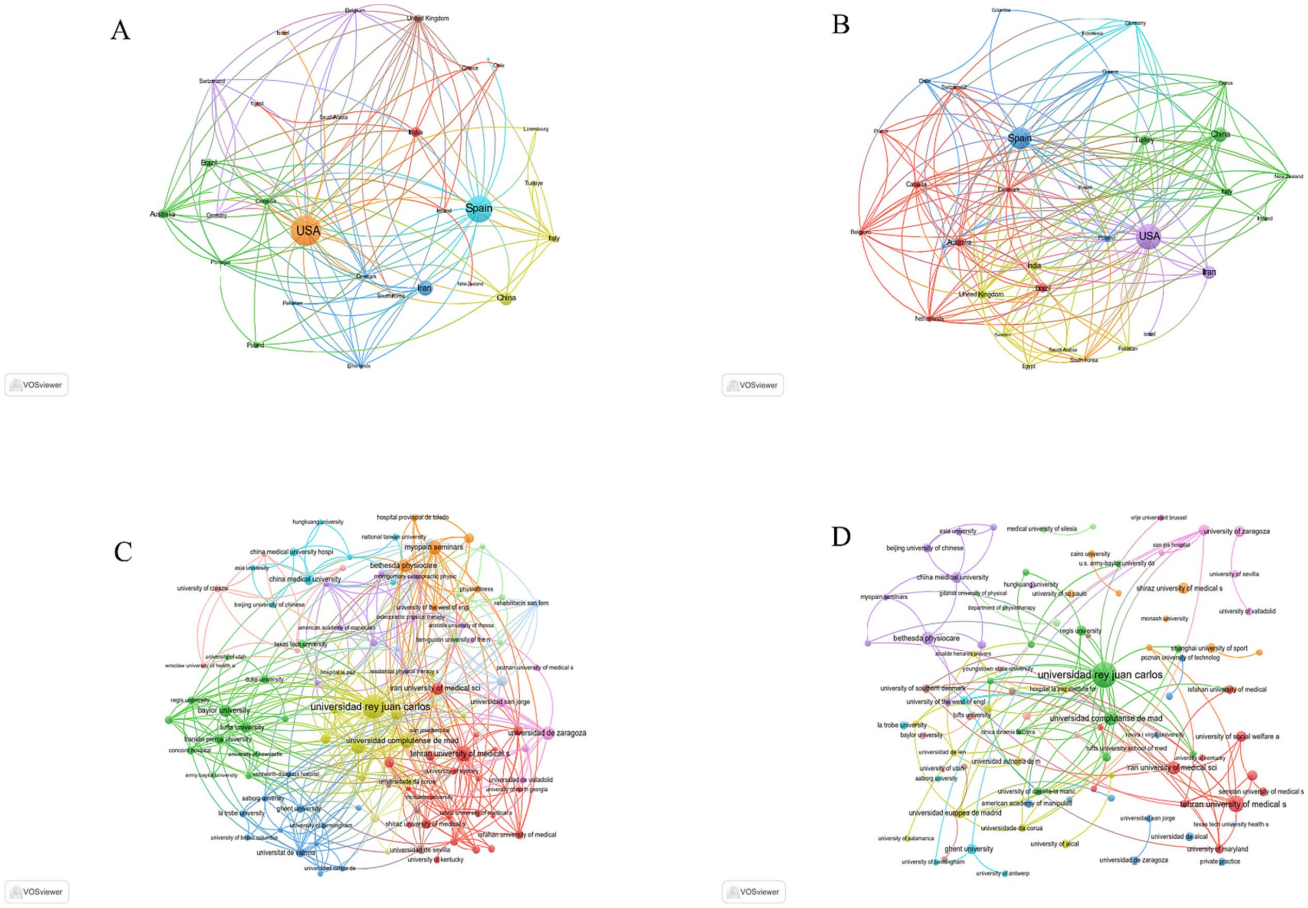
Global mapping of countries regions and institutions in DN for pain research from 2006 to 2025. **(A)** Geospatial collaboration network of countries based on WoSCC. **(B)** Geospatial collaboration network of countries based on Scopus. **(C)** Inter-institutional collaboration network derived from WoSCC. **(D)** Inter-institutional collaboration network derived from Scopus.

An analysis of authors’ countries revealed that, in the WoSCC database, the United States (*n* = 216) was the leading contributor in terms of publication volume, followed by Spain (*n* = 184), Iran (*n* = 92), China (*n* = 72), and India (*n* = 46). Additionally, 25.5% of publications from the United States and 29.9% from Spain involved international collaboration, as presented in Table 1 and Figure 2C. In the Scopus database, the United States (*n* = 231) again led, followed by Spain (*n* = 189), China (*n* = 99), Iran (*n* = 91), and Turkey (*n* = 49). Of these, 20.8% of U. S. publications and 32.3% of Spanish publications involved international collaboration, as shown in Table 1 and Figure 2D. Notably, both the United States and Spain demonstrated not only high publication output but also extensive international collaboration networks, as illustrated in Figures 3A,B. The prominence of these countries in DN research can be attributed to their well-established physical therapy systems, large professional workforce, comprehensive education and training infrastructure, and robust research policy support. Furthermore, the synergy between clinical application and mechanistic studies in these nations has fostered mature research ecosystems with significant international influence, thus propelling continued advancements in the field. Collaboration maps from both databases indicate that Universidad Rey Juan Carlos in Spain serves as a major hub of international cooperation (Figures 3C,D; Table 2).

**Table 1:** Most relevant countries by corresponding authors.

WoSCC				Scopus			
Country	Articles	MCP	MCP %	Country	Articles	MCP	MCP %
USA	216	55	25.5	USA	231	48	20.8
Spain	184	55	29.9	Spain	189	61	32.3
Iran	92	31	33.7	China	99	15	15.2
China	72	7	9.7	Iran	91	25	27.5
India	46	1	2.2	Turkey	49	0	0
Brazil	35	8	22.9	Brazil	46	7	15.2
Turkey	35	0	0	India	46	3	6.5
Canada	25	7	28	Canada	31	8	25.8
Australia	23	4	17.4	United Kingdom	30	7	23.3
Poland	23	8	34.8	Italy	23	6	26.1
Italy	18	5	27.8	Poland	23	7	30.4
Israel	15	2	13.3	Australia	22	5	22.7
United Kingdom	14	2	14.3	Israel	17	4	23.5
Pakistan	12	2	16.7	Pakistan	16	4	25
Belgium	11	5	45.5	Korea	11	4	36.4
Korea	10	3	30	Saudi Arabia	10	3	30
Saudi Arabia	7	2	28.6	Belgium	9	5	55.6
Denmark	6	2	33.3	Denmark	9	3	33.3
Egypt	5	1	20	Ireland	9	4	44.4
Greece	5	0	0	Indonesia	7	1	14.3

**Table 2:** Most relevant affiliations of the relationship on DN for Pain.

WoSCC				Scopus			
Affiliation	Articles (*n*)	Degree	Weighted Degree	Affiliation	Articles (*n*)	Degree	Weighted Degree
Universidad Rey Juan Carlos	93	55	231	Universidad Rey Juan Carlos	86	49	149
Tehran University of Medical Sciences	38	19	62	Tehran University of Medical Sciences	34	17	45
Universidad Complutense de Madrid	36	21	112	Universidad Complutense de Madrid	26	19	68
Universidad de Alcalá	30	24	59	Iran University of Medical Sciences	22	14	30
Myopain Seminars	28	19	87	Bethesda Physiocare	18	6	28
Universidad de Zaragoza	27	12	32	University of Social Welfare and Rehabilitation Sciences	15	9	13
Bethesda Physiocare	26	14	79	University of Zaragoza	15	12	15
Iran University of Medical Sciences	23	12	37	China Medical University	14	7	20
Baylor University	22	14	30	Shiraz University of Medical Sciences	14	4	4
Universidad Europea de Madrid	19	15	35	Ghent University	13	8	13
Universidad de Castilla-La Mancha	18	16	45	Shahid Beheshti University of Medical Sciences	13	7	14
Tufts University	17	16	56	Universidad Europea de Madrid	13	13	26
China Medical University	16	8	38	University of Valencia	13	17	24
Franklin Pierce University	15	14	37	Regis University	12	11	22
Shiraz University of Medical Sciences	14	5	5	Beijing University of Chinese Medicine	10	3	8
Shahid Beheshti University of Medical Sciences	13	9	20	Shanghai University of Sport	10	3	5
Shanghai University of Sport	13	3	5	Tufts University School of Medicine	10	8	27
Universidad CEU San Pablo	13	12	30	University of Maryland	10	12	27
Universidad San Jorge	13	6	15	American Academy of Manipulative Therapy Fellowship In Orthopaedic Manual Physical Therapy	9	2	7
Universitat de València	13	21	34	Franklin Pierce University	9	6	8

**Figure 4:**
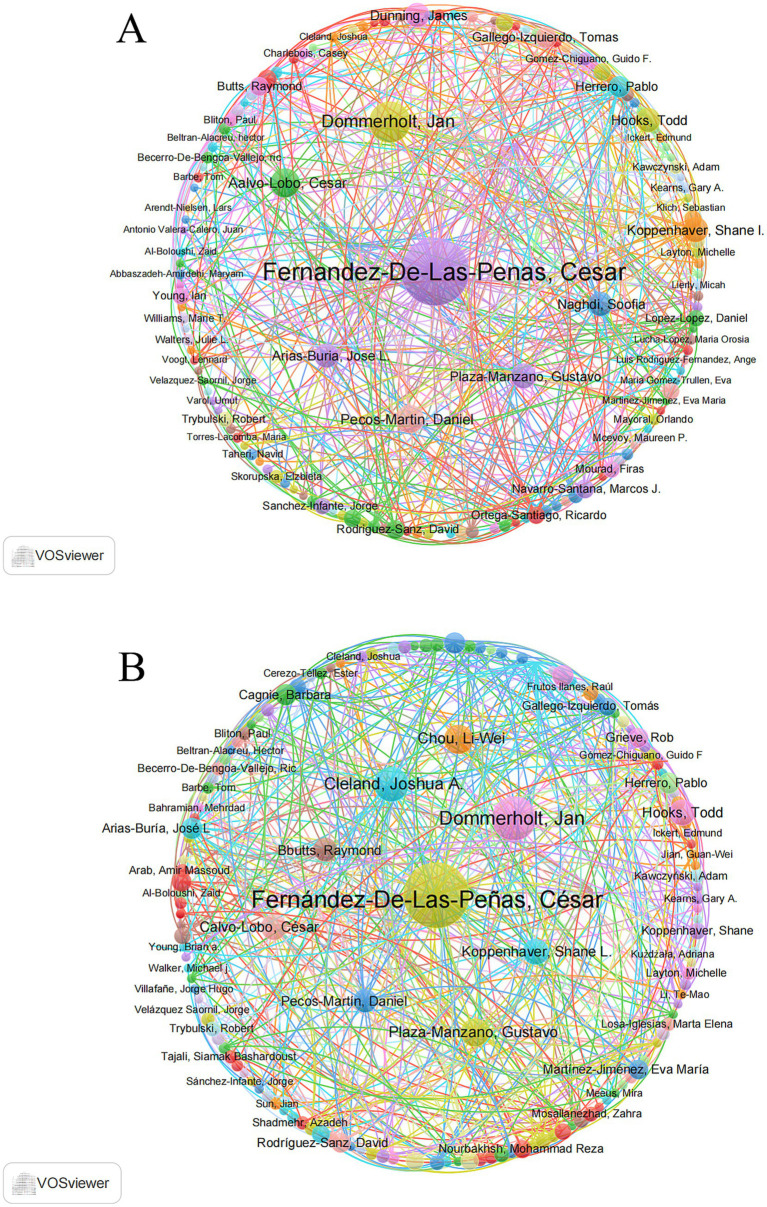
Co-authorship network analysis of contributing authors: **(A)** Network based on WoSCC; **(B)** network based on Scopus.

Author contribution analyses based on both the WoSCC and Scopus databases consistently identified Fernández-De-Las-Peñas C as the leading contributor to DN research in the field of pain, followed by Dommerholt J, Cleland JA, and Calvo-Lobo C. Notably, Fernández-De-Las-Peñas C ranked first in both databases, with fractionalized article counts of 10.46 in WoSCC and 12.57 in Scopus. This author also demonstrated strong academic impact, with H-indices of 23 (WoSCC) and 25 (Scopus), and G-indices of 37 (WoSCC) and 42 (Scopus). In addition, Fernández-De-Las-Peñas C showed a clear advantage in total citations (TC: 1,514 in WoSCC and 2,003 in Scopus) and total citations per year (TC per year: 26.10 in WoSCC and 28.61 in Scopus), further underscoring his prominent influence in this research domain (Figure 4; Table 3).

**Table 3:** Top 10 authors in DN for pain.

WoSCC							Scopus						
Author	Articles	Articles fractionalized	H-index	G-index	TC	TC per Year	Author	Articles	Articles fractionalized	H-index	G-index	TC	TC per Year
Fernández-De-Las-Peñas C	58	10.46	23	37	1,514	26.10	Fernández-De-Las-Peñas C	70	12.57	25	42	2003	28.61
Dommerholt J	33	8.16	8	14	231	7.00	Dommerholt J	32	8.62	15	21	496	23.62
Cleland JA	28	5.53	14	24	612	21.86	Cleland JA	28	5.56	15	27	731	26.11
Calvo-Lobo C	20	3.54	14	20	403	20.15	Calvo-Lobo C	21	3.66	14	20	416	20.80
Ansari NN	19	3.61	8	17	299	15.74	Arias-Buría JL	20	3.32	13	16	529	33.06
Arias-Buría JL	18	2.90	11	16	288	16.00	Chou L-W	17	3.66	11	14	500	35.71
Koppenhaver SL	17	2.77	11	16	281	16.53	Plaza-Manzano G	16	2.59	11	14	328	23.43
Plaza-Manzano G	16	2.50	11	16	465	29.06	Koppenhaver SL	15	2.43	10	23	572	17.88
Chou LW	15	3.35	8	15	429	28.60	Dunning J	14	2.85	10	12	417	34.75
Dunning J	15	2.83	8	15	271	18.07	Fernández-Carnero J	14	2.37	10	15	266	17.73

### 3.2 Journal analysis and visualization

To identify the most productive and most cited journals in the field of DN and pain, we utilized the Bibliometrix package in R. Graphical visualizations were created using the ggplot2 package. In addition, VOSviewer was employed to conduct journal co-citation analyses.

**Figure 5:**
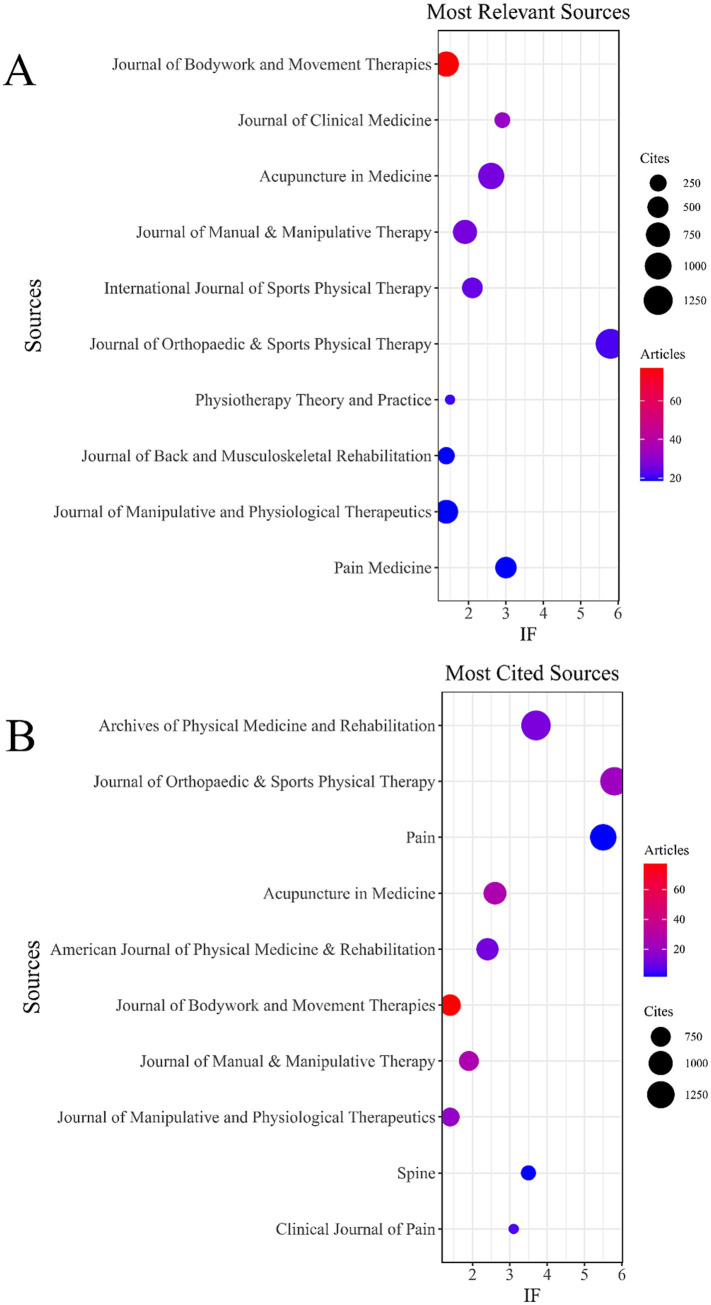
Journal with the largest number of articles published and the journal with the largest number of citations (WoSCC). **(A)** Journal with the largest number of articles published. **(B)** Journals with the largest number of citations.

In the WoSCC database, we analyzed 936 publications across 296 academic journals (see Supplementary material 1). As shown in Table 4 and Figure 5A, the *Journal of Bodywork and Movement Therapies* (*n* = 77, IF = 1.4) emerged as the most prolific journal, followed by the *Journal of Clinical Medicine* (*n* = 33, IF = 2.9), *Acupuncture in Medicine* (*n* = 27, IF = 2.6), *Journal of Manual & Manipulative Therapy* (*n* = 27, IF = 1.9), and the *International Journal of Sports Physical Therapy* (*n* = 25, IF = 2.1). Table 5 and Figure 5B highlight the most cited journals, including the *Archives of Physical Medicine and Rehabilitation* (*n* = 1,469, IF = 3.7), *Journal of Orthopaedic & Sports Physical Therapy* (*n* = 1,339, IF = 5.8), *Pain* (*n* = 1,156, IF = 5.5), *Acupuncture in Medicine* (*n* = 923, IF = 2.6), and the *American Journal of Physical Medicine & Rehabilitation* (*n* = 887, IF = 2.4).

**Table 4:** Top 10 journals with the most published articles.

WoSCC				Scopus		
Sources	Documents	Cites	IF (2024)	Sources	Documents	IF (2024)
Journal of Bodywork and Movement Therapies	77	831	1.4	Journal of Bodywork and Movement Therapies	84	1.4
Journal of Clinical Medicine	33	207	2.9	Journal of Clinical Medicine	37	2.9
Acupuncture in Medicine	27	923	2.6	Journal of Manual and Manipulative Therapy	29	1.9
Journal of Manual & Manipulative Therapy	27	750	1.9	Acupuncture in Medicine	26	2.6
International Journal of Sports Physical Therapy	25	469	2.1	Journal of Orthopaedic and Sports Physical Therapy	25	5.8
Journal of Orthopaedic and Sports Physical Therapy	22	1,339	5.8	Journal of Back and Musculoskeletal Rehabilitation	20	1.4
Physiotherapy Theory and Practice	21	107	1.5	Journal of Pain Research	20	2.5
Journal of Back and Musculoskeletal Rehabilitation	19	260	1.4	Pain Medicine	20	3
Journal of Manipulative and Physiological Therapeutics	19	697	1.4	Journal of Manipulative and Physiological Therapeutics	19	11.4
Pain Medicine	19	513	3	Evidence-Based Complementary and Alternative Medicine	17	0

**Table 5:** Top 10 journals with the most cited journals (WoSCC).

Sources	Cites	Documents	IF (2024)
Archives of Physical Medicine and Rehabilitation	1,469	13	3.7
Journal of Orthopaedic & Sports Physical Therapy	1,339	22	5.8
Pain	1,156	2	5.5
Acupuncture in Medicine	923	27	2.6
American Journal of Physical Medicine & Rehabilitation	887	12	2.4
Journal of Bodywork and Movement Therapies	831	77	1.4
Journal of Manual & Manipulative Therapy	750	27	1.9
Journal of Manipulative and Physiological Therapeutics	697	19	1.4
Spine	579	2	3.5
Clinical Journal of Pain	524	6	3.1

In the Scopus database, 1,149 publications were distributed across 420 academic journals (see Supplementary material 2). As shown in Table 4, the *Journal of Bodywork and Movement Therapies* (*n* = 84, IF = 1.4) was again the most productive, followed by the *Journal of Clinical Medicine* (*n* = 37, IF = 2.9), *Journal of Manual and Manipulative Therapy* (*n* = 29, IF = 1.9), *Acupuncture in Medicine* (*n* = 26, IF = 2.6), and the *Journal of Orthopaedic and Sports Physical Therapy* (*n* = 25, IF = 5.8).

**Figure 6:**
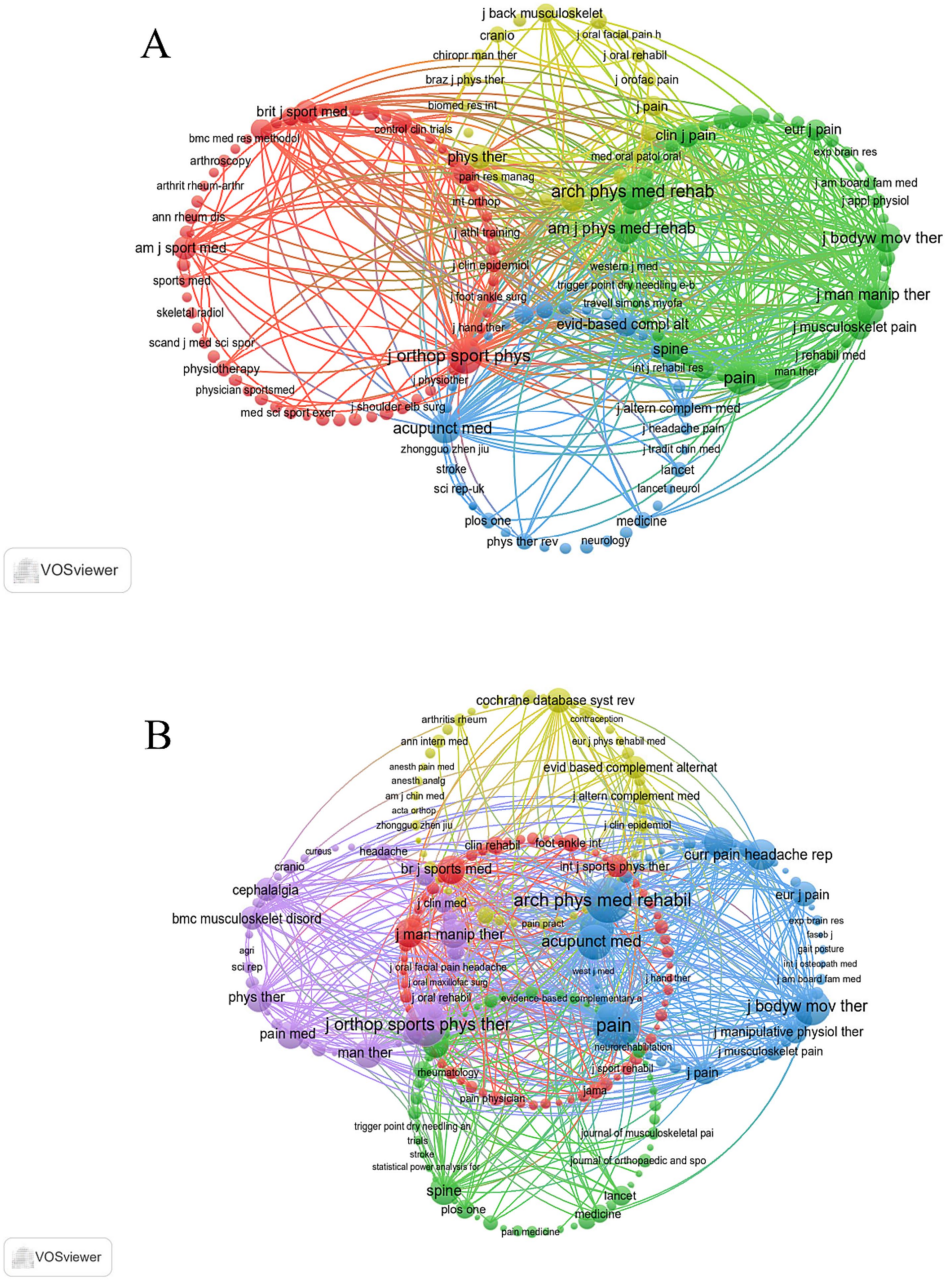
Co-cited journals related to DN for pain. **(A)** Journal co-citation network based on WoSCC. **(B)** Journal co-citation network based on Scopus.

Notably, co-citation analyses from both the WoSCC and Scopus databases (Figures 6A,B) revealed that the *Archives of Physical Medicine and Rehabilitation* serves as a central hub of scholarly collaboration.

Collectively, these findings underscore the pivotal roles of the *Journal of Bodywork and Movement Therapies* and the *Archives of Physical Medicine and Rehabilitation* in advancing research on DN and pain.

### 3.3 Most cited references and citation bursts

Using the Bibliometrix package in R, we identified the top 10 most cited articles in the field of DN and pain from the WoSCC database. Each of these articles has been cited over 178 times and is published across 8 different academic journals (see Table 6). The broad distribution of highly cited articles among multiple journals highlights the diversity of evidence sources in this field and underscores its interdisciplinary nature. Representative works include “Platelet-rich plasma as a treatment for patellar tendinopathy: a double-blind, randomized controlled trial,” “2018 Consensus statement on exercise therapy and physical interventions (orthoses, taping and manual therapy) to treat patellofemoral pain: recommendations from the 5th International Patellofemoral Pain Research Retreat, Gold Coast, Australia, 2017,” and “The Effectiveness of Platelet-Rich Plasma in the Treatment of Tendinopathy: A Meta-analysis of Randomized Controlled Clinical Trials.”

**Table 6:** Top 10 cited references related to the relationship on DN for Pain.

WoSCC			
Paper	DOI	Total citations	TC per year
DRAGOO JL, 2014, AM J SPORT MED	10.1177/0363546513518416	233	19.42
COLLINS NJ, 2018, BRIT J SPORT MED	10.1136/bjsports-2018-099397	225	28.13
FITZPATRICK J, 2017, AM J SPORT MED	10.1177/0363546516643716	216	24.00
KIETRYS DM, 2013, J ORTHOP SPORT PHYS	10.2519/jospt.2013.4668	214	16.46
CAGNIE B, 2013, CURR PAIN HEADACHE R	10.1007/s11916-013-0348-5	214	16.46
TOUGH EA, 2009, EUR J PAIN	10.1016/j.ejpain.2008.02.006	208	12.24
RHA DW, 2013, CLIN REHABIL	10.1177/0269215512448388	191	14.69
LIU L, 2015, ARCH PHYS MED REHAB	10.1016/j.apmr.2014.12.015	187	17.00
BIER JD, 2018, PHYS THER	10.1093/ptj/pzx118	184	23.00
GATTIE E, 2017, J ORTHOP SPORT PHYS	10.2519/jospt.2017.7096	178	19.78

Scopus			
Paper	DOI	Total citations	TC per year
BLANPIED PR, 2017, J ORTHOP SPORTS PHYS THER	10.2519/jospt.2017.0302	654	72.67
DRAGOO JL, 2014, AM J SPORTS MED	10.1177/0363546513518416	298	24.83
CORP N, 2021, EUR J PAIN	10.1002/ejp.1679	291	58.20
COLLINS NJ, 2018, BR J SPORTS MED	10.1136/bjsports-2018-099397	254	31.75
FITZPATRICK J, 2017, AM J SPORTS MED	10.1177/0363546516643716	239	26.56
CAGNIE B, 2013, CURR PAIN HEADACHE REP	10.1007/s11916-013-0348-5	235	18.08
TOUGH EA, 2009, EUR J PAIN	10.1016/j.ejpain.2008.02.006	231	13.59
RHA D-W, 2013, CLIN REHABIL	10.1177/0269215512448388	227	17.46
KIETRYS DM, 2013, J ORTHOP SPORTS PHYS THER	10.2519/jospt.2013.4668	225	17.31
DOMMERHOLT J, 2011, J MAN MANIP THER	10.1179/106698111×13129729552065	215	14.33

We also selected the top 10 most cited articles in the field of DN and pain from the Scopus database. Each of these articles has been cited more than 215 times and is distributed across 8 different academic journals (see Table 6). The most frequently cited publications on DN and pain include “Neck Pain: Revision 2017,” “Platelet-rich plasma as a treatment for patellar tendinopathy: a double-blind, randomized controlled trial,” and “Evidence-based treatment recommendations for neck and low back pain across Europe: A systematic review of guidelines.”

**Figure 7:**
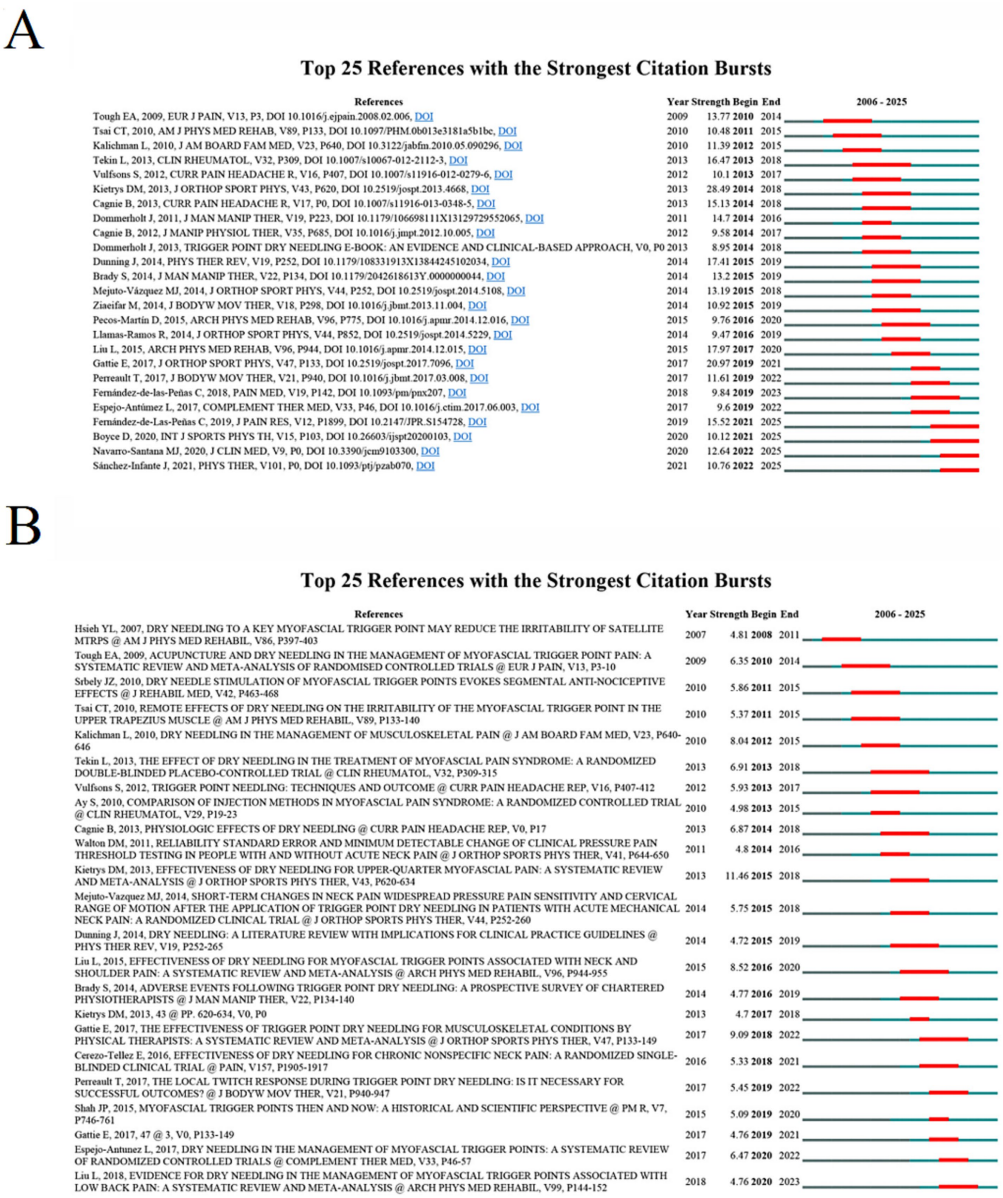
Top 25 references with the strongest citation bursts in DN for pain. **(A)** References from WoSCC, **(B)** references from Scopus.

To further explore the research frontiers and focal areas in DN and pain, we used CiteSpace to identify the top 25 references with the strongest citation bursts in the WoSCC database (see Figure 7A). The titles and DOIs of these references are listed in Supplementary material 3. The three most prominent citation bursts were: (1) “Effectiveness of dry needling for upper-quarter myofascial pain: a systematic review and meta-analysis” (strength: 28.49), (2) “The Effectiveness of Trigger Point Dry Needling for Musculoskeletal Conditions by Physical Therapists: A Systematic Review and Meta-analysis” (strength: 20.97), and (3) “Effectiveness of dry needling for myofascial trigger points associated with neck and shoulder pain: a systematic review and meta-analysis” (strength: 17.97). In addition, the three most recent citation bursts were: (1) “Is Dry Needling Applied by Physical Therapists Effective for Pain in Musculoskeletal Conditions? A Systematic Review and Meta-Analysis,” (2) “Effectiveness of Dry Needling for Myofascial Trigger Points Associated with Neck Pain Symptoms: An Updated Systematic Review and Meta-Analysis,” and (3) “Adverse events associated with therapeutic dry needling.”

Similarly, we used CiteSpace to identify the top 25 references with the strongest citation bursts in the Scopus database (see Figure 7B). The three most prominent citation bursts were: (1) “Effectiveness of dry needling for upper-quarter myofascial pain: a systematic review and meta-analysis” (strength: 11.46), (2) “The Effectiveness of Trigger Point Dry Needling for Musculoskeletal Conditions by Physical Therapists: A Systematic Review and Meta-analysis” (strength: 9.09), and (3) “Effectiveness of dry needling for myofascial trigger points associated with neck and shoulder pain: a systematic review and meta-analysis” (strength: 8.52). The three most recent citation bursts were: (1) “Trigger point dry needling for the treatment of myofascial pain syndrome: current perspectives within a pain neuroscience paradigm,” (2) “Effects of dry needling trigger point therapy in the shoulder region on patients with upper extremity pain and dysfunction: a systematic review with meta-analysis,” and (3) “Evidence for Dry Needling in the Management of Myofascial Trigger Points Associated With Low Back Pain: A Systematic Review and Meta-Analysis.”

Overall, based on the most frequently cited literature and citation burst analyses, we identified three major research directions in the field of DN and pain: (1) Evidence-based confirmation of clinical efficacy: Numerous randomized controlled trials (RCTs), systematic reviews, and meta-analyses have confirmed the clinical effectiveness of DN for MTrPs and related musculoskeletal pain, such as neck and shoulder pain, making it the central research hotspot in this field. (2) Physiological exploration of therapeutic mechanisms: Understanding the mechanisms underlying effects of DN is another key focus, with studies exploring its direct influence on peripheral tissues (e.g., muscle blood flow and oxygenation) and complex modulation of the peripheral and central nervous systems. (3) Standardization and safety assessment of clinical practice: The push toward standardization in clinical application constitutes the third major research trend, including the establishment of international consensus on diagnosis and procedures, evaluation of techniques such as local twitch responses, and systematic assessment of adverse events associated with DN interventions.

### 3.4 Keyword clusters and thematic evolution

**Figure 8:**
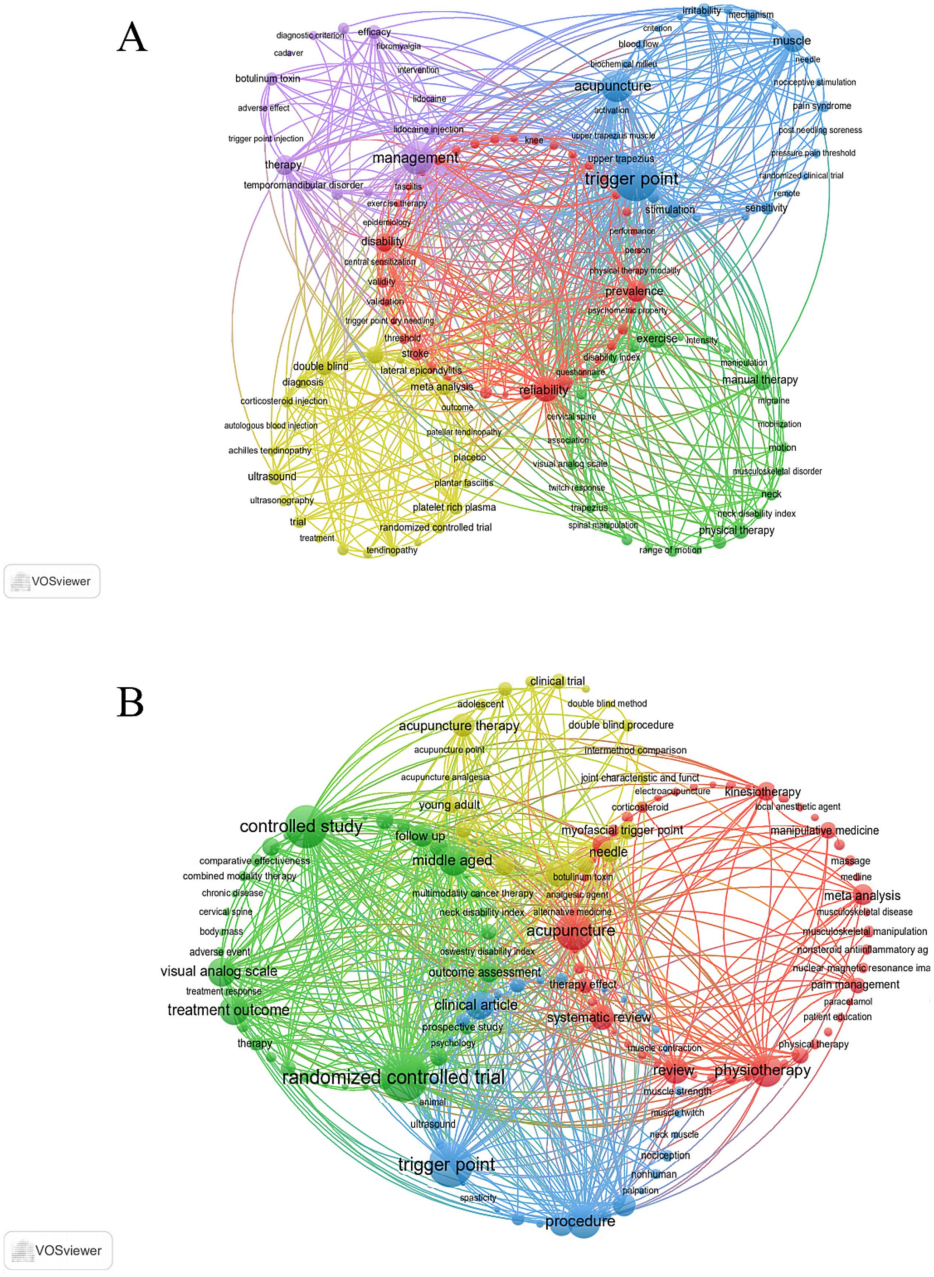
Keyword co-occurrence map of publications in DN for pain. **(A)** Keyword co-occurrence analysis based on WoSCC. **(B)** Keyword co-occurrence analysis based on Scopus.

Keyword clustering is crucial for quickly grasping the primary research themes and directions within a given field. In our study, VOSviewer identified 2,296 keywords in the WoSCC database. Table 7 lists the top 20 keywords occurring more than 45 times, highlighting the core research focuses. Among them, “Trigger Point” (*n* = 487) was the most frequent, followed by “Management” (*n* = 274), “Acupuncture” (*n* = 239), “Reliability” (*n* = 157), and “Muscle” (*n* = 123). Cluster analysis revealed five distinct color-coded clusters in Figure 8A. (1) Clinical Measurement and Assessment in Rehabilitation (red dots, 40 keywords), with core terms such as “reliability,” “prevalence,” and “disability,” focuses on clinical assessment in rehabilitation medicine. This hotspot centers around the reliability and validity of various scales and questionnaires (e.g., Modified Ashworth Scale) applied to specific diseases such as post-stroke spasticity and osteoarthritis, aiming to establish a robust psychometric foundation for clinical outcome evaluation. (2) Physical Interventions for Musculoskeletal Pain (green dots, 28 keywords), includes terms such as “exercise,” “manual therapy,” and “physical therapy,” and represents studies on physical interventions for musculoskeletal pain. This cluster compares the effects of various physical therapies (e.g., manual therapy, electroacupuncture) on conditions like neck and shoulder pain through clinical trials, focusing on indicators such as range of motion and pain levels. (3) Pathophysiology of MTrPs (blue dots, 27 keywords), is centered on “trigger point” and includes terms like “mechanism,” “biochemical milieu,” and “spontaneous electrical activity.” This reflects a focus on the pathophysiological mechanisms underlying MTrPs. Research in this area explores the formation and activation of trigger points, and their neural and biochemical correlates with pain and sensitivity, from a basic science perspective. (4) Evidence-Based Evaluation of Interventions for Tendinopathies (yellow dots, 24 keywords), includes terms like “randomized controlled trial,” “injection,” and “tendinopathy,” highlighting evidence-based comparative research within the context of dry needling. This hotspot is characterized by the use of meta-analyses and high-level clinical trials to systematically compare dry needling with other mainstream interventions, including platelet-rich plasma (PRP), corticosteroid injections, and extracorporeal shock wave therapy, for tendinopathies such as tennis elbow and plantar fasciitis, often with ultrasound assistance for diagnosis and treatment guidance. (5) Management of Complex Pain Syndromes (purple dots, 17 keywords), with terms like “management,” “efficacy,” and “systematic review,” focuses on the comprehensive management of complex pain syndromes. This hotspot systematically evaluates high-level evidence on various interventions (e.g., botulinum toxin, lidocaine injection) for intractable disorders such as temporomandibular disorder and fibromyalgia. All keywords are listed in Supplementary material 4.

**Table 7:** Top 20 keywords related to the relationship on DN for Pain.

WoSCC		Scopus	
Words	Occurrences	Words	Occurrences
Trigger Point	487	Randomized Controlled Trial	481
Management	274	Trigger Point	441
Acupuncture	239	Controlled Study	424
Reliability	157	Acupuncture	341
Muscle	123	Procedure	295
Prevalence	107	Physiotherapy	274
Therapy	92	Treatment Outcome	242
Disability	80	Visual Analog Scale	234
Exercise	80	Clinical Article	232
Manual Therapy	80	Review	211
Double Blind	71	Systematic Review	184
Rehabilitation	69	Analgesia	172
Injection	66	Acupuncture Therapy	171
Physical Therapy	62	Follow Up	169
Stimulation	54	Pathophysiology	169
Meta Analysis	53	Range of Motion	169
Ultrasound	53	Needle	148
Irritability	47	Meta Analysis	147
Upper Trapezius	47	Major Clinical Study	134
Platelet Rich Plasma	45	Outcome Assessment	134

Using VOSviewer, 5,664 keywords were identified from the Scopus database. Table 7 lists the top 20 keywords occurring more than 134 times, showcasing key research focuses. Among these, “Randomized Controlled Trial” (*n* = 481) was most frequent, followed by “Trigger Point” (*n* = 441), “Controlled Study” (*n* = 424), “Acupuncture” (*n* = 341), and “Procedure” (*n* = 295). Cluster analysis revealed four distinct color-coded clusters in Figure 8B. (1) Evidence-Based Review of Pain Management (red dots, 43 keywords) includes terms such as “acupuncture,” “physiotherapy,” and “systematic review,” representing an evidence-based research direction centered on dry needling and its systematic comparison with other pain interventions. This cluster is characterized by the extensive use of systematic reviews and meta-analyses to comprehensively evaluate the clinical effectiveness and analgesic outcomes of dry needling in comparison with manual therapy, injection therapies such as corticosteroids and platelet-rich plasma, and other physiotherapy modalities. (2) Clinical Trial Methodology and Outcome Assessment (green dots, 30 keywords), includes terms such as “randomized controlled trial” and “controlled study,” focusing on clinical research design and outcome assessment. This hotspot emphasizes rigorous study design (e.g., single−/double-blind trials), objective outcome measures (e.g., visual analog scales, quality of life, disability indices), and long-term follow-up to provide a standardized framework for evaluating treatment outcomes. (3) Diagnostic Imaging and Pathophysiological Mechanisms of Myofascial Pain (blue dots, 25 keywords), centers on “trigger point” and explores its pathophysiology and diagnostic methods. This research relies heavily on technologies such as “ultrasound/echography” and “electromyography” to visualize and quantify skeletal muscle physiology, strength, and trigger point characteristics (e.g., muscle twitch response), and investigates their relationship with nociception. (4) Clinical Efficacy of DN for Specific Myofascial Pain (yellow dots, 15 keywords), includes terms like “acupuncture therapy,” “needle,” and “myofascial trigger point.” This cluster focuses on the effect of DN on specific muscles (e.g., trapezius), often employing rigorous “double blind procedure” trials to compare different techniques, with special attention to functional improvements such as articular range of motion. All keywords are listed in Supplementary material 5.

**Figure 9:**
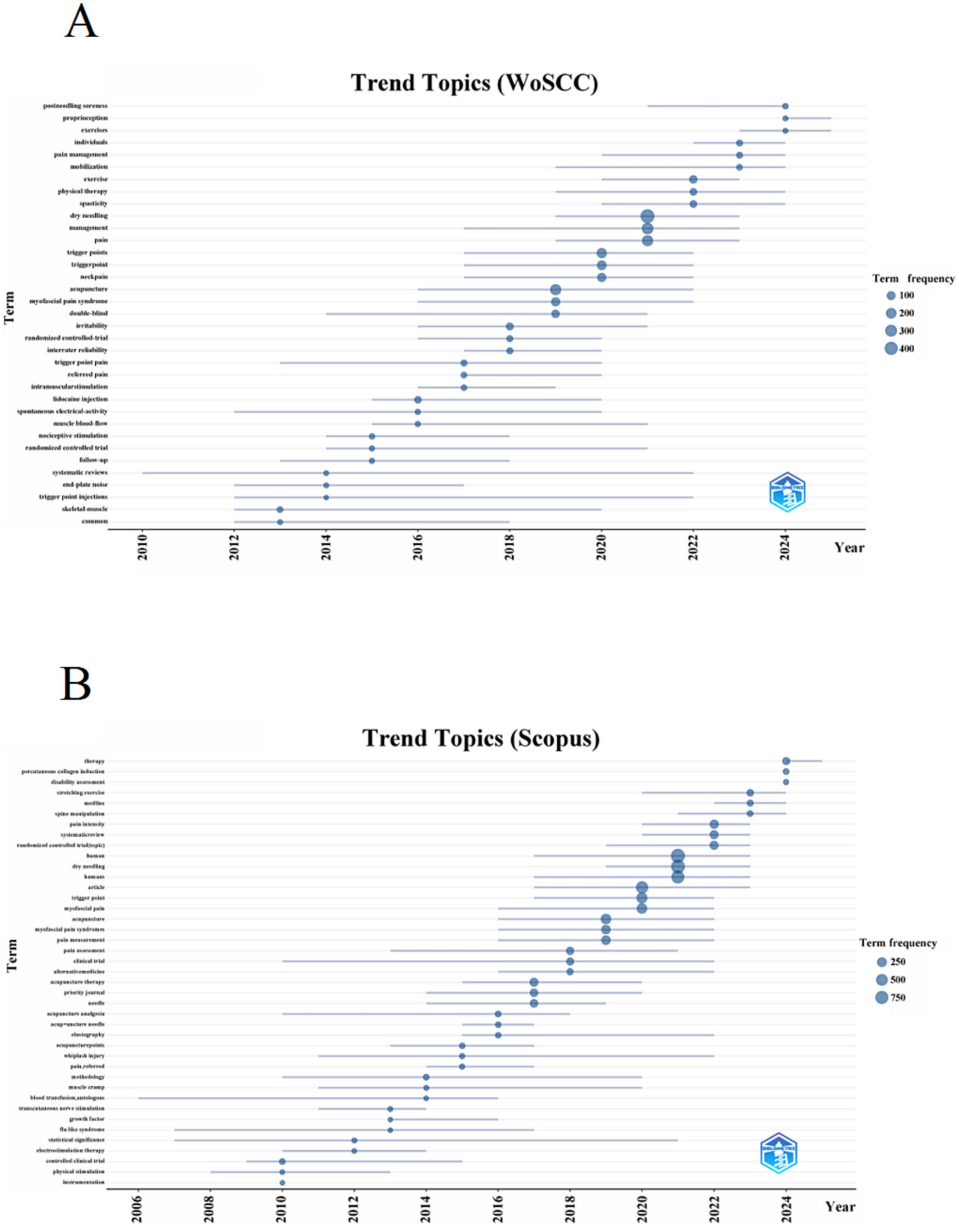
Trend topics on DN for pain. **(A)** Trend topics based on WoSCC. **(B)** Trend topics based on Scopus.

In addition, to forecast future trends in the field, a dynamic thematic evolution map was constructed using the Bibliometrix package in the R environment. As shown in Figure 9A (WoSCC data), early research (pre-2016) focused on fundamental mechanisms. Keywords during this period included “end-plate noise” and “spontaneous electrical activity,” with scholars aiming to elucidate the pathophysiological basis of trigger points, and beginning preliminary randomized controlled trials (RCTs) to assess therapeutic efficacy. In the mid-stage (2017–2020), research shifted toward clinical application and evidence consolidation. Conditions such as “myofascial pain syndrome” and “neck pain” became central topics, and there was a marked increase in methodological rigor, including emphasis on “interrater reliability” and “double-blind” protocols, indicating field maturation. In the recent stage (2021 to present), the frontier has shifted toward integrated management strategies. Core themes now include combinations of “dry needling” with “exercise” and “physical therapy,” along with the emergence of refined research areas such as “postneedling soreness” and “proprioception,” suggesting a future increasingly oriented toward integrative, patient-centered approaches. In the Scopus data (Figure 9B), early research (pre-2016) focused on foundational methodology and initial efficacy validation. During this phase, “controlled clinical trials” compared acupuncture with other modalities such as “transcutaneous nerve stimulation,” often targeting specific conditions like “whiplash injury,” and beginning exploration of phenomena like “pain, referred.” In the mid-stage (2017–2020), research entered a phase of rapid development and evidence consolidation. “Trigger point” and “myofascial pain syndromes” became the dominant focus. The academic community sought to standardize “pain assessment” and “pain measurement,” while introducing new techniques such as “elastography” to investigate underlying mechanisms. “Acupuncture therapy” and “needle” became high-frequency terms, indicating a central focus on the intervention itself. In the recent stage (2021 to present), research has evolved toward terminological precision, high-level evidence, and integrated treatments. “Dry needling” has emerged as the dominant term, accompanied by a surge in “systematic reviews” and high-quality “randomized controlled trials.” The research paradigm is expanding from single interventions to comprehensive rehabilitation approaches, combining DN with “stretching exercise” and “spine manipulation.” The emergence of novel techniques such as “percutaneous collagen induction” and growing focus on “disability assessment” suggest the field is progressing toward a new phase of technological innovation and function-oriented practice.

Overall, keyword co-occurrence and thematic evolution analyses indicate that DN research has exhibited a clear trend toward increasing maturity and diversification over time. Since approximately 2015, the volume of DN–related publications has increased markedly, with research priorities gradually shifting from explorations of the pathophysiological mechanisms of MTrPs to studies oriented toward clinical application and evidence-based practice. Research themes have evolved from predominantly mechanistic investigations to the generation of high-level evidence, exemplified by RCTs and systematic reviews. In parallel, keywords related to physical therapy, exercise, and rehabilitation outcomes have appeared with increasing frequency, suggesting that DN is being progressively integrated into comprehensive rehabilitation programs and multimodal pain management strategies. Collectively, these trends indicate that DN research has transitioned from relatively isolated intervention studies to a more standardized, collaborative, and clinically translatable research paradigm.

### 3.5 Synthesis of research hotspots

In summary, through extensive analyses—including citation bursts, keyword frequency, clustering, and thematic evolution—we identified the primary research hotspots in the field of DN and pain. These hotspots converge around four key directions: (1) The clinical efficacy of DN for musculoskeletal pain and its evidence-based positioning. This area systematically confirms the therapeutic effectiveness of DN across various musculoskeletal disorders through high-quality evidence and comparative evaluations against other modalities such as manual therapy and exercise. (2) The pathophysiological mechanisms and visualization techniques in DN for pain relief. This research direction leverages tools like ultrasound and electromyography to investigate the biochemical and electrophysiological features of MTrPs, as well as the neuroregulatory mechanisms activated by DN. (3) Clinical trial methodology and outcome assessment in DN research. This focus aims to enhance the methodological rigor of RCTs and to develop reliable and valid assessment tools for standardized outcome evaluation. (4) Standardization of clinical practice and safety risk management in DN for pain. This area seeks to establish international consensus on diagnostic criteria and procedural protocols for trigger point treatment, and to systematically assess adverse events to inform safe and effective clinical implementation.

## 4 Discussion

### 4.1 Major findings and knowledge structure of DN research for pain

Based on data from both the WoSCC and Scopus databases, this study provides a systematic analysis of the global research landscape of DN for pain from 2006 to 2025. The results demonstrate a sustained and accelerating growth in publication output, suggesting that DN has evolved from a relatively niche technique into an important research focus within pain management and rehabilitation medicine (24, 25).

From the perspective of knowledge structure, DN research exhibits a high degree of concentration at the country, institutional, and author levels. The United States and Spain consistently occupy central positions in terms of publication output and international collaboration networks, reflecting the deep integration of DN into physical therapy education systems, clinical guidelines, and research agendas in these countries. At the author level, a limited number of highly influential scholars form the academic core of the field and have made pivotal contributions to methodological refinement and the accumulation of evidence-based knowledge.

In addition, DN research shows clear trends toward increasing maturity and diversification over time. Over the past two decades, the volume of related publications has increased markedly, with research themes gradually shifting from investigations of the pathophysiological mechanisms of MTrPs to high-quality studies oriented toward clinical application and evidence-based medicine (24). This transition is reflected in bibliometric indicators through distinct evolutions in keywords and themes: early studies primarily focused on mechanistic exploration, whereas recent research has been dominated by evidence generation in the form of RCTs and systematic reviews. Concurrently, keywords related to physical therapy, exercise, and rehabilitation outcomes have appeared with increasing frequency, indicating that DN is being progressively integrated into comprehensive rehabilitation programs and multimodal pain management strategies.

Overall, the development of DN research over the past two decades has been characterized by continuous growth in publication volume, a highly concentrated knowledge structure, progressive thematic maturation, diversification of research paradigms, and enhanced clinical translatability. These trends not only reflect the rising academic attention to DN as a physical intervention, but also suggest that the field is gradually establishing a more standardized, collaborative, and evidence-oriented research framework, thereby providing a solid foundation for future high-quality clinical trials and mechanistic studies.

### 4.2 Comparison with previous bibliometric and review studies

Previous bibliometric studies on pain-related needling or non-pharmacological interventions have often analyzed DN within broader categories of acupuncture techniques or physical therapy, without clearly distinguishing it as an independent intervention (19, 26, 27). While such approaches are useful for capturing overarching research trends at a macro level, they may obscure the specific research characteristics and developmental pathways of DN. In contrast, the present study explicitly treats DN as an independent research entity and conducts parallel analyses using both the WoSCC and Scopus databases, thereby methodologically reducing the potential bias associated with reliance on a single database.

In terms of research conclusions, earlier reviews and bibliometric analyses have primarily emphasized the clinical application of DN in musculoskeletal pain or focused on its potential analgesic mechanisms, with relatively limited attention to its overall knowledge structure and evolutionary trajectory (7, 28, 29). By integrating multiple dimensions—including publication trends, author and institutional networks, and keyword evolution—the present study demonstrates that DN research has not developed in isolation. Instead, it has progressed simultaneously along clinical and mechanistic research pathways, exhibiting an intertwined and co-evolving pattern over time.

### 4.3 Clinical implications of emerging research hotspots

Hotspot analysis indicates that recent DN research for pain has increasingly focused on RCTs, systematic reviews, pain intensity, pressure pain thresholds, and function-related outcome measures. This pattern suggests that the evaluative framework of the field is moving toward greater standardization and quantification, with DN being progressively incorporated into evidence-based research paradigms.

Notably, DN frequently co-occurs with keywords related to exercise training, physical therapy, and rehabilitation interventions. From both research design and clinical application perspectives, this co-occurrence implies that DN is more often investigated within integrated treatment frameworks rather than as a standalone intervention. From a bibliometric standpoint, this trend reflects a growing research emphasis on multimodal and combined intervention strategies in pain management.

### 4.4 Neurophysiological significance and directions for mechanistic research

Throughout the thematic evolution of the field, neurophysiological mechanisms underlying the analgesic effects of DN have remained a persistent research focus. Keyword clustering analysis indicates that studies related to MTrPs, bioelectrical activity, ultrasound imaging, and electromyographic monitoring have occupied prominent positions across different developmental stages, forming an essential knowledge base for mechanistic research in this domain.

Existing studies have primarily explored the mechanisms of DN from the perspectives of local tissue responses, electrophysiological changes, and neural modulation (30–33). With the introduction of imaging and electrophysiological techniques, some investigations have attempted to objectively characterize trigger point features and post-intervention changes following DN (34–36). These studies form relatively stable thematic clusters within bibliometric networks, indicating sustained academic interest in mechanistic research within the DN field.

These persistently investigated mechanistic research directions indicate that the field of dry needling continues to explore and contribute to a deeper understanding of the neurophysiological basis of pain. Further elucidation of these neuromodulatory mechanisms in future studies may help to more comprehensively clarify the therapeutic pathways of dry needling in the management of neuromuscular pain.

### 4.5 Study limitations

This study conducted a systematic bibliometric analysis of the scale, hotspots, and developmental trends of research on DN interventions for pain based on the WoSCC and Scopus databases. While this approach facilitates a macroscopic understanding of the knowledge structure and research evolution of the field, several inherent limitations should be considered when interpreting the results.

First, only two major international citation databases—WoSCC and Scopus—were included. Although both databases are widely recognized and offer substantial advantages for citation analysis and bibliometric research, differences in journal coverage, disciplinary classification systems, and citation indexing strategies may introduce database indexing bias. Consequently, some studies related to DN for pain, particularly those published in regional journals or specialized rehabilitation and physical therapy databases, may not have been captured, potentially affecting the comprehensiveness of the analysis. Second, despite the use of a systematic search strategy, search bias cannot be entirely excluded. Bibliometric analyses depend on predefined search terms, and variations in terminology related to DN techniques, pain classifications, and study contexts may have resulted in the omission of some high-quality studies. In addition, inconsistencies in keyword standardization across journals may further limit literature coverage. Third, analyses of highly cited literature and citation bursts are inherently influenced by citation bias. Highly cited publications are often older articles, reviews, or guidelines published in high-impact journals, whereas recently published high-quality studies or those appearing in lower-impact journals may be underrepresented. Moreover, reciprocal citation practices within specific academic groups may amplify the visibility of certain research directions without fully reflecting overall trends in the field. Fourth, only English-language publications were included, introducing potential language bias. Although English dominates international scientific communication and WoSCC and Scopus effectively reflect mainstream global research trends, high-quality studies conducted in non-English-speaking countries—particularly in China, where DN is increasingly applied in rehabilitation practice—may not have been fully captured. This limitation constrains a comprehensive representation of the field’s global knowledge base and reflects the dominance of English-language journals in international academic dissemination.

Despite these limitations, the dual-database analytical approach employed in this study provides a relatively robust overview of the overall developmental trends, research hotspots, and potential frontiers in DN research for pain, offering a valuable macroscopic reference and evidence base for future investigations.

### 4.6 Implications for future research

The findings of this bibliometric analysis suggest that research on DN for pain can be further advanced along established trajectories. From a methodological perspective, future bibliometric studies may adopt more refined analytical techniques, such as topic modeling, to more comprehensively characterize thematic structures and their temporal evolution. Within current clinical research frameworks, greater consistency in outcome measure selection would enhance comparability across studies and improve the interpretability of evidence synthesis. In addition, the continued incorporation of mechanism-related outcome measures into established study designs may facilitate a deeper understanding of the links between experimental and clinical research. Overall, these directions are consistent with the emerging trends toward methodological standardization and evidence integration observed in DN research for pain.

## 5 Conclusion

This study conducted a systematic bibliometric analysis of the global research landscape on DN for pain from 2006 to 2025 based on the WoSCC and Scopus databases. The dual-database design offers clear methodological advantages by enhancing data coverage and reducing bias associated with reliance on a single database, thereby enabling a more comprehensive and reliable depiction of research trends, core contributors, and thematic evolution within this field.

The results demonstrate a sustained increase in publication output related to DN for pain over the study period, accompanied by a gradual diversification of research themes. Analyses at the levels of countries, institutions, authors, and journals identified relatively stable patterns of research contribution and collaboration, as well as core journals that play a pivotal role in knowledge dissemination within the field. Keyword co-occurrence and thematic evolution analyses further revealed that research hotspots have expanded from early exploratory topics toward more systematic clinical and methodological domains, reflecting the progressive maturation of the research framework.

For clinicians, the identification of core journals, highly productive countries, and relatively stable research themes provides a clear structure for systematically navigating the literature on DN for pain. These findings may facilitate more efficient identification of influential studies and improve understanding of the disease types and clinical application scenarios that have been most frequently investigated.

For researchers, the clearly delineated thematic clusters and their temporal evolution illustrate a developmental trajectory in which research priorities have shifted from mechanistic exploration toward clinical application and methodological standardization. This structured knowledge mapping helps identify areas with relatively concentrated evidence, as well as topics where research remains fragmented or is undergoing transition, thereby offering more targeted guidance for future study design.

Future research may build on the current findings by incorporating artificial intelligence–assisted bibliometric mapping and topic modeling approaches to enhance the granularity of theme identification and the capacity for knowledge integration. In addition, further strengthening the standardization of outcome measures and the integration of mechanism-related research within existing frameworks may contribute to the continued optimization and advancement of DN in pain management.

## Data Availability

The datasets presented in this study can be found in online repositories. The names of the repositories and accession numbers can be found in the article/Supplementary material.

## Footnotes

1
http://www.bibliometrix.org
